# Acute exercise impacts AhR and PD-1 levels of CD8^+^ T-cells—Exploratory results from a randomized cross-over trial comparing endurance versus resistance exercise

**DOI:** 10.1007/s00421-020-04552-w

**Published:** 2020-11-19

**Authors:** Alexander Schenk, Niklas Joisten, David Walzik, Christina Koliamitra, Daria Schoser, Wilhelm Bloch, Philipp Zimmer

**Affiliations:** 1 Institute for Sport and Sport Science, TU Dortmund University, Dortmund, Germany; 2 Institute for Cardiovascular Research and Sports Medicine, German Sports University Cologne, Cologne, Germany; 3 Institute of Movement Therapy and Movement Oriented Prevention and Rehabilitation, German Sports University, Cologne, Germany

**Keywords:** Acute exercise, T-cell, AhR, PD-1, Kynurenine

## Abstract

**Purpose:**

The programmed cell death protein 1 (PD-1) has become a promising target in cancer immunotherapy. PD-1 expression of CD8^+^ T-cells may be increased via the exploitation of aryl hydrocarbon receptor (AhR) signaling with kynurenine (KYN) as a ligand. Since exercise affects KYN metabolism, we exploratory investigated the influence of acute exercise bouts on AhR and PD-1 levels of CD8^+^ T-cells.

**Method:**

In this study, 24 healthy males (age: 24.6 ± 3.9 years; weight 83.9 ± 10.5 kg; height: 182.4 ± 6.2 cm) completed a single bout of endurance (EE) and resistance exercise (RE) in a randomly assigned order on separate days. Blood samples were drawn before (t0), after (t1), and 1 h after (t2) both conditions. T-cell populations, the level of cytoplasmic AhR, and surface PD-1 were assessed by flow cytometry.

**Results:**

T-cell populations changed over time, indicated by an increase in the absolute numbers of CD3^+^ lymphocytes after EE (*p* < .001) and RE (*p* = .036) and in PD-1^+^ CD8^+^ T-cells after EE (*p* = .021). Proportions of T-cell populations changed only after EE (t0–t2: *p* = .029; t1-t2: *p* = .006). The level of cytoplasmic AhR decreased immediately after exercise in both exercise conditions (EE: *p* = .009; RE: *p* = .036). The level of surface PD-1 decreased 1 h after EE (*p* = .005).

**Conclusion:**

We analyzed the level of surface PD-1 and cytoplasmic AhR following acute physical exercise for the first time. Especially EE was observed to impact both AhR and PD-1 levels, undermining its role as the AhR-PD-1 axis modulator. These results provide new insights into the impact of exercise on AhR-signaling, which could potentially be relevant for various chronic diseases.

**Electronic supplementary material:**

The online version of this article (10.1007/s00421-020-04552-w) contains supplementary material, which is available to authorized users.

## Introduction

Programmed cell death protein 1 (PD-1) is a major checkpoint in T-cell function, with remarkable results as an immunotherapeutic target in a vast number of malignancies (Ai et al. 2020). PD-1 is an inhibitory receptor that promotes apoptosis and functional exhaustion upon ligand binding (Shi et al. 2019). In the tumor microenvironment, tumor cells induce the PD-1 expression of CD8^+^ cytotoxic T-cells, thereby leading to reduced T-cell function and increased tumor surveillance (Liu et al. 2018).

As described by Liu et al. (2018) this signaling cascade of tumor cells depends on the secretion of kynurenine (KYN), a metabolite of the essential amino acid tryptophan (TRP). KYN is synthesized by the indoleamine 2,3-dioxygenase (IDO) using TRP as a substrate (Opitz et al. 2011). Tumor cells have been described to express high levels of IDO (Opitz et al. 2011; Uyttenhove et al. 2003; Munn und Mellor 2016) and secrete KYN into the tumor microenvironment (Liu et al. 2018). CD8^+^ T-cells ingest the secreted KYN that in turn acts as a ligand for the intracellular aryl hydrocarbon receptor (AhR) (Labadie et al. 2019; Liu et al. 2018). After ligand binding, the AhR translocates from the cytoplasm into the nucleus, acting as a transcription factor. AhR controls the expression of various genes involved in complex processes, among others also inflammation (Martin et al. 2020). Besides genes such as *CYP1A1, CYP1B1, IDO* or the AhR repressor gene (Rothhammer und Quintana 2019), AhR also induces gene expression of PD-1 (Liu et al. 2018). KYN is further metabolized to kynurenic acid (KA) or quinolinic acid (QA), the former representing an AhR-ligand as well (DiNatale et al. 2010; Martin et al. 2020). Therefore, KA might be of equal interest as a signaling molecule in immune regulation.

Exercise is known to have a profound influence on the human immune system (Walsh et al. 2011). Acute bouts of exercise are characterized by an increased lymphocytosis during and immediately after exercise, with the most pronounced increases in natural killer (NK) and CD8^+^ T-cells (Gustafson et al. 2017). Gustafson et al. (2017) observed increases in the relative amount of PD-1^+^ T-cells after exercise. Whether this increase in PD-1^+^ T-cells is due to changes in T-cell homeostasis or changes in PD-1 gene expression has not been addressed in this study. In a recent study by Wadley et al. (2020) the increase of PD-1^+^ CD8^+^ T-cells was acknowledged. The authors describe further subsets of the CD8^+^ T-cell pool showing the highest increase in PD-1^+^ central memory CD8^+^ T-cells that also showed an increase in PD-1 expression after a single bout of exercise. Both investigations used an endurance exercise bout and so far, no investigations using resistance exercise were performed. However, the physical activity recommendations of the world health organization (World Health Organization 2010) comprise a weekly amount of endurance and resistance exercise. Also in clinical settings, primarily endurance and resistance exercise are used, but especially for resistance exercise, the knowledge of underlying mechanisms for benefits is scarce. In general, endurance exercise is thought to have a higher impact on immune cell homeostasis, cytokine levels, and the KYN-pathway (Joisten et al. 2020; Schlagheck et al. 2020), whereas there is a considerable lack of knowledge about the effects of resistance exercise.

The KYN-AhR axis may play a key role in exercise-induced alterations of PD-1^+^ T-cell. As reviewed by Joisten et al. (2020), several studies observed an influence of acute exercise on KYN pathway metabolites. Investigating different exercise modalities, elevated levels of KYN and KA were frequently observed immediately after exercise (Joisten et al. 2020; Koliamitra et al. 2019; Strasser et al. 2016). Since both metabolites KYN and KA serve as AhR ligands, the question arises if exercise-induced changes in these metabolites impact intracellular AhR levels and the level of PD-1 on the cell surface. Therefore, we performed a secondary analysis of the study published by Joisten et al. (2020) and investigated in an exploratory manner whether a single bout of endurance or resistance exercise impacts the cytoplasmic AhR levels and the level of PD-1 on the cell surface of CD8^+^ T-cells. Since the impact of acute exercise on the KYN pathway towards KA has been described to be more pronounced following endurance than resistance exercise (Joisten et al. 2020) and both KYN and KA are endogenous ligands of the AhR (DiNatale et al. 2010; Martin et al. 2020), we expect greater changes induced by endurance exercise. Due to the activation-induced translocation of AhR, we hypothesize that endurance exercise results in a greater decrease in the cytoplasmatic AhR level, whereas surface PD-1 levels show a greater increase.

## Methods

### Study design

The present study is a secondary analysis of a previously published study. A detailed description of the study design and associated procedures is presented elsewhere (Joisten et al. 2020).

In brief, 24 healthy young males (mean age ± SD: 24.6 ± 3.9 years, mean VO_2_peak ± SD: 48.3 ± 7.4 ml/kg/min and a mean total strength value ± SD of 101.9 ± 16.2 kg) were recruited for participation in a randomized cross-over study comparing single exercise bouts of endurance exercise (EE) and resistance exercise (RE). Our aim was to investigate the acute effects of clinically applicable resistance (hypertrophy) and endurance (continuous moderate to vigorous aerobic) exercise in a time-matched intervention design. On a separate testing day, participants performed a baseline assessment of strength and endurance capacity to define individual intensities for both exercise bouts. To assess endurance capacity, an incremental exercise test on a stationary bicycle ergometry was performed until exhaustion. To assess strength capacity, participants conducted a one-repetition maximum (1-RM) test on exercise machines (Cybex International) in the following order: chest press, lat pull, leg curl, leg extension, back extension. The total strength value was calculated as the mean of all five 1RM scores. Both exercise sessions were conducted on separate testing days with at least 48 h in between each day. Both sessions lasted 50 min and were conducted between 8 and 11 AM. Nutritional intake was kept similarly before both sessions. Participants were allowed to drink water ad libitum. The room temperature was 21 °C. The EE session consisted of 60% peak power output achieved in the baseline testing for 45 min following a 5 min warm-up period. The RE session consisted of four sets of 8–10 repetitions at 70% of 1RM at each machine. Blood samples were collected immediately before (*t*0), immediately after (*t*1), and 1 h after (*t*2) each bout of exercise. In the present study, blood samples were analyzed using flow cytometry to determine intracellular AhR levels and the level of PD-1 on the cell surface of CD8^+^ T-cells.

### Blood sample preparation

EDTA blood was used for a blood count (Sysmex, Norderstedt, Germany), followed by isolation of peripheral blood mononuclear cells (PBMCs) via density gradient centrifugation using a lymphocyte separation medium (LSM; PromoCell, Heidelberg, Germany). Blood samples were diluted with PBS, carefully layered on top of the LSM, and centrifuged for 30 Min at 800×g. After extraction of the PBMC containing interphase, the cells were washed with PBS and centrifuged for 10 Min at 800×g. The remaining cell pellet was resuspended in Recovery™ cell culture freezing medium (Thermo Fischer Scientific, Waltham, MA, USA) and frozen at  – 80 °C overnight. Cells were stored at  – 150 °C until flow cytometry analyses were conducted.

### Flow cytometry

Using flow cytometry, the proportions and numbers of CD3^+^ T-cells, CD8^+^ T-cells, and PD-1^+^ CD8^+^ T-cells were analyzed. Cells were thawed and suspended in PBS for flow cytometry. Thereafter, cells were stained with fluorochrome coupled antibodies (BD, Heidelberg, Germany) for CD3 (PE-Cy7), CD8 (APC-Cy7), and CD279/PD-1 (APC) for 30 min at room temperature (RT) according to the manufacturer´s information. Afterwards cells were fixated and remaining erythrocytes lysed with whole blood lysing solution (Thermo Fischer Scientific, Waltham, MA, USA) for 10 min. Subsequently, cells were washed three times with Perm/Wash Buffer (BD, Heidelberg, Germany) for cell permeabilization. Next, cells were suspended in Perm/Wash buffer for staining of intracellular AhR (PE) for 30 min at 4 °C. Thereafter cells were washed and suspended in Sheath solution (BD, Heidelberg, Germany) for measurement. Flow cytometry analyses were performed on a BD FACSArray (BD, Heidelberg, Germany). For visualization and gating FACSDiva Software (BD, Heidelberg, Germany) was used. For data analysis, the percentages of CD3^+^ T-cells, CD8^+^ T-cells and PD-1^+^ CD8^+^ T-cells and mean fluorescence intensity (MFI) of PD-1 and AhR within the CD8^+^ T-cell population was exported. Furthermore, absolute numbers of T-cell populations were calculated using the lymphocyte numbers from the blood count.

### Statistics

Data are presented as mean ± SEM. Outliers were identified by z-transformation. All outliers (z-score > 3) were removed from statistical analyses. Separate univariate baseline-adjusted analysis of covariance (ANCOVA) models was used to test for statistical main effects. Greenhouse–Geisser correction was applied if sphericity was violated (Mauchly’s test). In case of statistical main effects for time and/or interaction (time*group) Bonferroni corrected pairwise comparisons were applied to determine within and/or between-group differences. Statistical analyses were conducted using SPSS 25 (IBM, Armonk, NY, USA). The level of significance was set at *p* equal or less 0.05.

## Results

**Fig. 1:**
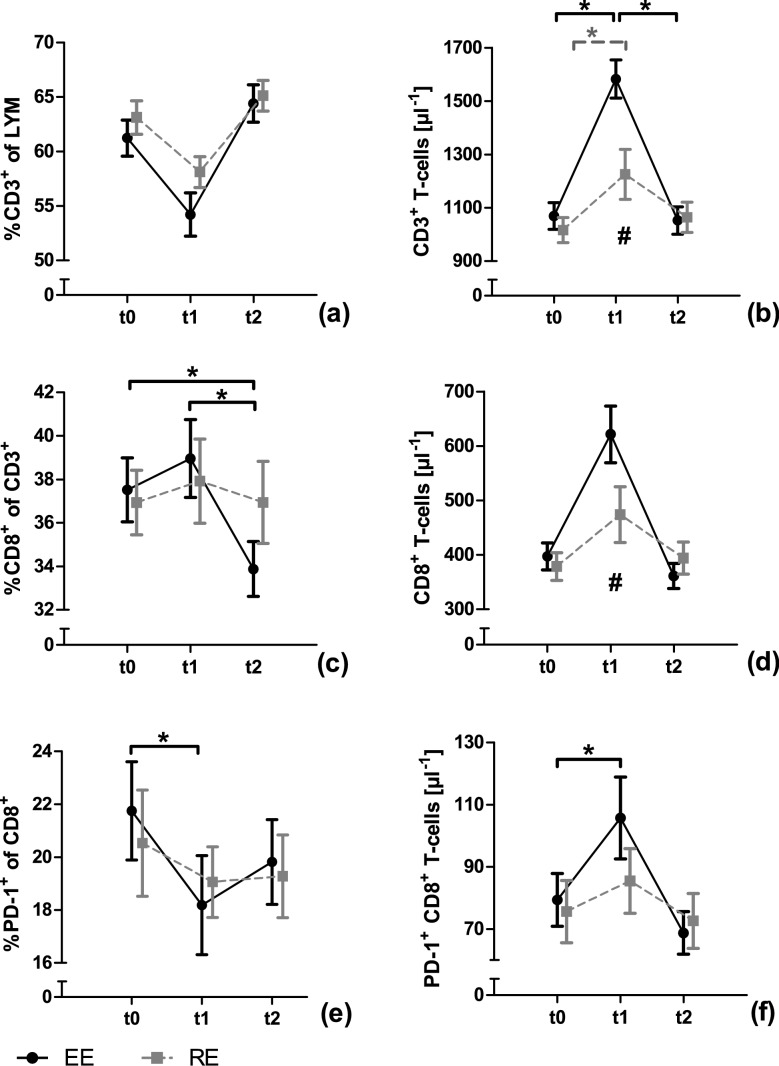
Changes in T-cell numbers and proportions in response to a single bout of endurance (EE) or resistance exercise (RE). T-cells are presented before (t0), immediately after (t1) and 1 h after (t2) a single bout of EE and RE, respectively. T-cells are presented in the subsets of CD3^+^ T-cells (**a**, **b**), CD8^+^ T-cells (**c**, **d**) and PD1^+^ CD8^+^ T-cells (**e**, **f)**. Data are presented as mean ± SEM. Statistically significant effects are marked with a * for time effects and # for interaction effects

**Fig. 2:**
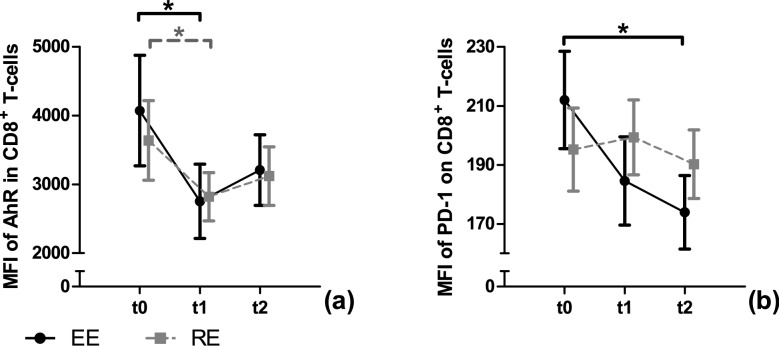
Level of AhR and PD-1 of CD8^+^ T-cells in response to a single bout of endurance (EE) or resistance exercise (RE). The mean fluorescence intensities (MFI) of cytoplasmatic AhR (**a**) and surface PD-1 (**b**) are used as a relative surrogate marker of protein level. Data are presented as mean ± SEM. Statistically significant effects were marked with a * for time effects and # for interaction effects

Absolute and relative changes of T-cell subsets are presented in Fig. 1. Surface PD-1 and cytoplasmic AhR levels are shown in Fig. 2. Detailed ANCOVA results for all outcome measurements are provided in Table 1. Raw data is presented in supplement 1.

**Table 1:** Detailed ANCOVA results of all outcome measures

	Group size (N)		ANCOVA time			ANCOVA interaction (time × group)		
	EE	RE	*p*	*F*	df	*p*	*F*	df
CD3^+^ (#)	21	22	0.030*	4.262	1.433	0.001*	9.210	1.433
CD3^+^ (% of LYM)	22	22	0.082	2.583	1.632	0.199	1.676	1.632
CD8^+^ (#)	21	22	0.452	0.680	1.318	0.015*	5.501	1.318
CD8 + (% of CD3^+^)	22	22	0.028*	4.147	1.609	0.080	2.782	1.609
PD-1^+^ CD8^+^ (#)	20	21	0.042*	3.687	1.541	0.159	1.963	1.541
PD-1^+^ CD8^+^ (% of CD8^+^)	22	22	0.006*	5.858	1.734	0.629	0.421	1.734
MFI PD-1 on CD8^+^	19	22	0.001*	8.377	1.705	0.292	1.240	1.705
MFI PD-1 on PD-1^+^ CD8^+^	20	22	0.028*	3.730	2.000	0.339	1.996	2.000
MFI AhR on CD8^+^	21	21	0.001*	7.352	2.000	0.902	0.104	2.000

*EE* endurance exercise, *RE* resistance exercise, *p* *p*-value, *F* *F*-value, *df *degrees of freedom, # cell counts (μl^−1^), % cell proportions, *significant ANCOVA result (*p* ≤ 0.05), *LYM* lymphocytes, *CD* cluster of differentiation, *MFI* mean fluorescence intensity, *PD-1* programmed cell death protein 1, *AhR* aryl hydrocarbon receptor

### Cell numbers and proportions

Neither EE nor RE statistically affected the proportions of CD3^+^ T-cells. The absolute number of CD3^+^ T-cells showed a statistically significant time and interaction effect. Post-hoc analysis revealed a statistical increase in CD3^+^ T-cell numbers from t0 to t1 (*p* < 0.001) and a statistical decrease from t1 to t2 (*p* < 0.001) for EE. For RE, a statistical increase in CD3^+^ T-cell numbers from t0 to t1 (*p* = 0.036) was observed. A statistical interaction effect was observed for CD3^+^ T-cells at t1, with numbers being higher in EE compared to RE (*p* = 0.036). The proportions of CD8^+^ T-cells showed a statistically significant time effect with a decrease from t0 to t2 *(p* = 0.029) and from t1 to t2 (*p* = 0.006) in EE. For RE, no change in CD8^+^ T-cell proportions was observed. For absolute numbers of CD8^+^ T-cells, the ANCOVA revealed no time but a statistical interaction effect with higher numbers in EE than in RE at t1 (*p* = 0.044). The proportions of PD-1^+^ CD8^+^ T-cells decreased significantly over time from t0 to t1 in EE (*p* = 0.003), while in RE neither time nor interaction effects were observed. Absolute numbers of PD-1^+^ CD8^+^ T-cells changed statistically significant overtime in EE from t0 to t1 (*p* = 0.021) and from t1 to t2 (*p* = 0.003). In RE, no time or interaction effects were found for PD-1^+^ CD8^+^ T-cells.

### Level of PD-1 and AhR

For the MFI of PD-1, a statistically significant time effect with a decrease from t0 to t2 (*p* = 0.005) was observed in EE. In RE, no time or interaction effects were revealed. The ANCOVA showed a significant time but no interaction effect for the MFI of AhR. Post-hoc tests showed a significant decrease for the MFI of AhR from t0 to t1 in EE (*p* = 0.009) and RE (*p* = 0.036).

## Discussion

Acute exercise is known to have a profound impact on immune cell homeostasis. Using an exploratory approach, we investigated whether acute bouts of exercise impact cytoplasmic AhR levels and PD-1 expression on CD8^+^ T-cells. Especially EE was observed to impact both AhR levels and PD-1 expression. These novel results might be of major relevance for exercise-induced changes in immune function in general but also for exercise as a supportive treatment strategy in oncologic and other clinical settings.

The proportion of PD-1^+^ cells within the CD8^+^ T-cell population decreased after EE. In line with results from Gustafson et al. (2017) and Wadley et al. (2020) the absolute number of PD-1^+^ CD8^+^ T-cells increased after EE. Therefore, EE is capable of influencing PD-1^+^ T-cell homeostasis and may also influence T-cell function. The comparison of proportions and absolute numbers revealed a relatively higher influx of PD-1^−^ CD8^+^ T-cells than PD-1^+^ CD8^+^ T-cells, thereby potentially influencing the functional properties of the CD8^+^ T-cell pool. The increase of absolute numbers of PD-1^+^ CD8^+^ T-cells is, at least in part, due to an increase of the CD3^+^ T-cell population. The influence of a single bout of exercise on T-cells and their subsets was already shown in 1998 by Mazzeo et al. (1998), also indicating an increase of the CD3^+^ T-cells and subsequent increases of their subsets. Regarding cell surface PD-1 levels there is a decrease from baseline to 1 h after EE, while relative and absolute values of PD-1^+^ CD8^+^ T-cells return to baseline within this timeframe. This indicates a cellular change of CD8^+^ T-cells, marked by a lower surface PD-1 expression per cell, potentially reducing PD-1 associated CD8^+^ T-cell suppression. Furthermore, PD-1 expression is associated with T-cell exhaustion and reduced functionality (Wherry und Kurachi 2015). As described in the review of Simpson (2011) exercise mobilizes preferentially senescent and exhausted T-cells to undergo apoptosis in peripheral tissues. This is explained to make “immunological space” for naïve T-cells and an improved T-cell function. Therefore, this could be an explanation for the decrease in surface levels of PD-1.

In contrast to EE, RE does not influence the PD-1 levels on CD8^+^ T-cells, indicating that EE is a more potent stimulus for alterations in PD-1 surface expression. To the best of our knowledge, this is the first study investigating the influence of RE on PD-1 levels and comparing EE with RE. This may be of relevance in the context of PD-1-based immunotherapies. PD-1 is a critical immune checkpoint regulating the antigen response of T- and B-cells and has shown very promising results as an immunotherapeutic target (Ai et al. 2020). In the last decades, physical activity has gained more and more attention as supportive therapy for cancer patients to reduce side effects, but also to improve therapy success as such (Ashcraft et al. 2019). However, the knowledge of underlying mechanisms is still sparse. Since the PD-1 pathway is frequently targeted in immunotherapy and modifiable by exercise, PD-1 signaling might be a potential mechanism for exercise-induced benefits in cancer therapy. Regarding PD-1 blockade, an exercise-induced decrease of surface PD-1 on CD8^+^ T-cells by EE may support immunotherapy by reducing PD-1 levels on CD8^+^ T-cells.

Results from analyses of the tumor microenvironment revealed the regulation of PD-1 expression by the intracellular AhR. KYN is secreted by tumor cells and is further described to serve as a ligand for the cytoplasmic AhR of CD8^+^ T-cells, thereby inducing PD-1 expression and immune surveillance (Liu et al. 2018). Interestingly, several studies have shown increases in KYN and KA immediately after acute exercise (Joisten et al. 2020; Koliamitra et al. 2019; Strasser et al. 2016). Since not only KYN, but also KA is a potent AhR ligand it can be suspected that exercise affects PD-1 expression by KYN/KA-AhR signaling. Here, we were able to show that the levels of cytoplasmic AhR decreased immediately after EE and RE. After KYN/KA binding to AhR, the activated form translocates from the cytoplasm into the nucleus (Ikuta et al. 2004), which might explain reduced cytoplasmic AhR levels. However, during sample preparation, a saponin-based cell permeabilization was used. Saponin is able to permeabilize the cell membrane but not the nuclear membrane (Goldenthal et al. 1985). Thus, the measurement of AhR levels only captures the inactive cytoplasmic form and does not provide information on the activation of AhR. To the best of our knowledge, it is not described whether or not the cytoplasmic AhR levels correlate with those in the nucleus. Therefore, it remains unclear if a decrease in cytoplasmic AhR levels is due to an activation or protein degradation. The non-activated AhR resides in the cytoplasm bound to a multi-protein complex, consisting of heat shock proteins (HSPs) and chaperones, protecting non-activated AhR from nuclear import and protein degradation (Esser 2016). Since HSP expression is altered by acute exercise (Connolly et al. 2004), this could potentially impact the multi-protein complex leading to (i) nuclear transport of non-activated AhR or (ii) enhanced AhR degradation.

In the context of the AhR-PD-1 axis, a reduction of cytoplasmic AhR due to activation could be expected to result in an increase of surface PD-1 levels. Nevertheless, the surface levels of PD-1 within the CD8^+^ T-cell population decreases after EE. Thus, two possible explanations are hypothesized. On the one hand, EE could interfere with the multi-protein complex leading to an unspecific decrease in cytoplasmic AhR levels. On the other hand, EE could activate other pathways regulating PD-1 expression in an AhR-independent manner. In contrast to EE, RE did only show minor effects on AhR-PD-1 axis, which potentially could be due to a less pronounced KYN-pathway activation. Further research approaches are needed to examine the response of the AhR-PD-1 axis to acute EE in a more detailed manner.

### Strengths and limitations

This study investigated the influence of acute exercise on the AhR-PD-1 axis, exposing a new promising mechanism of exercise immunology. Especially since PD-1 is a popular target in cancer immunotherapy, this mechanism might play a pivotal role for exercise-induced benefits in supportive cancer therapy and beyond. As a secondary analysis of a previously published study, no power calculation for the investigated endpoints was performed. Nevertheless, relatively large sample size was analyzed in a randomized cross-over study. In the present study, we did not include a passive control group. Due to the acute exercise protocol with all measurement time points within 2 h, a passive control group is not a requirement, however, it should be mentioned as a limitation. Furthermore, the comparability of the used EE and RE sessions is questionable. While accurate matching for energy expenditure or workload would increase the experimental comparability of the interventions, the practical relevance and application in clinical or recreational settings would have been of marginal significance. Therefore, we decided to investigate two time-matched interventions with high practical relevance. Albeit limiting the transferability of the results to other populations, such as women, older persons or diseased populations, we examined a homogenous sample to obtain consistent results. Since an explorative approach was chosen to provide first insights into this promising topic, AhR was only measured in the cytoplasm, rather than analyzing AhR-levels in the cytoplasm and nucleus.

## Conclusion

For the first time, an influence of acute exercise bouts on the amount of cytoplasmic AhR and surface PD-1 expression of CD8^+^ T-cells was observed. Especially, EE affects the AhR-PD-1 axis, which represents a promising mechanism for exercise immunology. Considering previous results, exercise-induced alterations of the KYN pathway might represent potential mediators for altered AhR and PD-1 levels. Further studies are needed to investigate the influence of exercise on the AhR-PD-1 axis in detail, especially considering potential mediation by the KYN pathway as well as different immune cell populations.

## Electronic supplementary material

Below is the link to the electronic supplementary material.

Supplementary file1 (DOCX 18 KB)

## Abbreviations

1-RM: One-repetition maximum
AhR: Aryl hydrocarbon receptor
EE: Endurance exercise
IDO: Indoleamine 2,3-dioxygenase
KA: Kynurenic acid
KYN: Kynurenine
MFI: Mean fluorescence intensity
NK-cell: Natural killer cells
PBMCs: Peripheral blood mononuclear cells
PD-1: Programmed cell death protein 1
QA: Quinolinic acid
RE: Resistance exercise
RT: Room temperature
SD: Standard deviation
SEM: Standard error of the mean
TRP: Tryptophan
