# Supplementary material for: Acute exercise impacts AhR and PD-1 levels of CD8+ T-cells—Exploratory results from a randomized cross-over trial comparing endurance versus resistance exercise
Source: Eur J Appl Physiol. 2020 Nov 19;121(2):637–44. doi: 10.1007/s00421-020-04552-w (PMC7862188; doi:10.1007/s00421-020-04552-w)
Supplement: Supplementary file 1 — Supplementary file1 (DOCX 18 KB) [file 421_2020_4552_MOESM1_ESM.docx]

Table 2. Raw data of all outcome measures separated by exercise session.

|  | EE | | | RE | | |
| --- | --- | --- | --- | --- | --- | --- |
|  | t_0_ | t _1_ | t _2_ | t _0_ | t _1_ | t _2_ |
| CD3^+^ (#) | 1069.04 ± 230.1 | 1582.78 ± 328.2 | 1052.62 ± 235.36 | 1016.76 ± 221.85 | 1225.84 ± 440.02 | 1064.77 ± 264.68 |
| CD3^+^ (% of LYM) | 61.23 ± 7.79 | 54.21 ± 9.3 | 64.41 ± 8.07 | 63.13 ± 7.2 | 58.12 ± 6.63 | 65.13 ± 6.62 |
| CD8^+^ (#) | 397.33 ± 114.17 | 621.48 ± 238.97 | 361.47 ± 105.92 | 378.65 ± 118.36 | 473.93 ± 239.92 | 394.31 ± 137.75 |
| CD8+ (% of CD3^+^) | 37.51 ± 6.89 | 38.96 ± 8.38 | 33.88 ± 5.94 | 36.94 ± 6.95 | 37.92 ± 9.08 | 36.94 ± 8.84 |
| PD-1^+^ CD8^+^ (#) | 79.35 ± 38.02 | 105.72 ± 58.69 | 68.72 ± 30.72 | 75.6 ± 45.72 | 85.45 ± 47.6 | 72.61 ± 40.55 |
| PD-1^+^ CD8^+^ (% of CD8^+^) | 21.75 ± 8.73 | 18.19 ± 8.79 | 19.82 ± 7.53 | 20.53 ± 9.44 | 19.06 ± 6.26 | 19.28 ± 7.33 |
| MFI PD-1 on CD8^+^ | 212 ± 71.97 | 184.58 ± 65.26 | 173.95 ± 54.39 | 195.18 ± 66.12 | 199.32 ± 59.43 | 190.23 ± 54.56 |
| MFI PD-1 on PD-1^+^ CD8^+^ | 728.35 ± 96.82 | 713.85 ± 109.58 | 654.4 ± 137.55 | 665.73 ± 68.36 | 713.86 ± 128.29 | 684.55 ± 103.59 |
| MFI AhR on CD8^+^ | 4070.38 ± 3677.42 | 2753.86 ± 2479.93 | 3207.62 ± 2346.47 | 3637.86 ± 2641.62 | 2819.76 ± 1607.52 | 3119.52 ± 1939.5 |

Data is displayed as mean ± SD. EE endurance exercise; RE resistance exercise; t_0_ pre-exercise; t_1_ immediately after exercise; t_2_ 1 hour after exercise; # cell counts (μl^-1^); % cell proportions; LYM lymphocytes; CD cluster of differentiation; MFI mean fluorescence intensity; PD-1 programmed cell death protein 1; AhR aryl hydrocarbon receptor
